# Challenges of HIV Self-Test Distribution for Index Testing When HIV Status Disclosure Is Low: Preliminary Results of a Qualitative Study in Bamako (Mali) as Part of the ATLAS Project

**DOI:** 10.3389/fpubh.2021.653543

**Published:** 2021-05-19

**Authors:** Sokhna Boye, Seydou Bouaré, Odette Ky-Zerbo, Nicolas Rouveau, Arlette Simo Fotso, Marc d'Elbée, Romain Silhol, Mathieu Maheu-Giroux, Anthony Vautier, Guillaume Breton, Abdelaye Keita, Anne Bekelynck, Alice Desclaux, Joseph Larmarange, Dolorès Pourette

**Affiliations:** ^1^Centre Population et Développement (Ceped), Institut de Recherche pour le Développement (IRD), Université de Paris, Inserm, Paris, France; ^2^Institut de Pédagogie Universitaire, Bamako, Mali; ^3^TransVIHMI (IRD, Université de Montpellier, INSERM), Montpellier, France; ^4^Department of Global Health and Development, Faculty of Public Health and Policy, London School of Hygiene and Tropical Medicine, London, United Kingdom; ^5^UK Medical Research Council Centre for Global Infectious Disease Analysis, School of Public Health, Imperial College London, London, United Kingdom; ^6^Department of Epidemiology, Biostatistics, and Occupational Health, School of Population and Global Health, McGill University, Montreal, QC, Canada; ^7^Solthis, Dakar, Sénégal; ^8^Solthis, Paris, France; ^9^Département qualité sécurité et sécurité biologique, Institut National de Santé Publique, Bamako, Mali; ^10^Programme PAC-CI, ANRS Research Site, Treichville University Hospital, Abidjan, Côte d'Ivoire; ^11^Institut de Recherche pour le Développement, Transvihmi (IRD, INSERM, Montpellier University), Montpellier, France; ^12^CRCF, Dakar, Sénégal

**Keywords:** HIV self-testing, index testing, knowledge of HIV status, HIV status disclosure, Mali, partners of PLHIV, people living with HIV, screening

## Abstract

**Context:** The rate of HIV status disclosure to partners is low in Mali, a West African country with a national HIV prevalence of 1.2%. HIV self-testing (HIVST) could increase testing coverage among partners of people living with HIV (PLHIV). The *AutoTest-VIH, Libre d'accéder à la connaissance de son Statut* (ATLAS) program was launched in West Africa with the objective of distributing nearly half a million HIV self-tests from 2019 to 2021 in Côte d'Ivoire, Mali, and Senegal. The ATLAS program integrates several research activities. This article presents the preliminary results of the qualitative study of the ATLAS program in Mali. This study aims to improve our understanding of the practices, limitations and issues related to the distribution of HIV self-tests to PLHIV so that they can offer the tests to their sexual partners.

**Methods:** This qualitative study was conducted in 2019 in an HIV care clinic in Bamako. It consisted of (i) individual interviews with eight health professionals involved in the distribution of HIV self-tests; (ii) 591 observations of medical consultations, including social service consultations, with PLHIV; (iii) seven observations of peer educator-led PLHIV group discussions. The interviews with health professionals and the observations notes have been subject to content analysis.

**Results:** HIVST was discussed in only 9% of the observed consultations (51/591). When HIVST was discussed, the discussion was almost always initiated by the health professional rather than PLHIV. HIVST was discussed infrequently because, in most of the consultations, it was not appropriate to propose partner HIVST (e.g., when PLHIV were widowed, did not have partners, or had delegated someone to renew their prescriptions). Some PLHIV had not disclosed their HIV status to their partners. Dispensing HIV self-tests was time-consuming, and medical consultations were very short. Three main barriers to HIVST distribution when HIV status had not been disclosed to partners were identified: (1) almost all health professionals avoided offering HIVST to PLHIV when they thought or knew that the PLHIV had not disclosed their HIV status to partners; (2) PLHIV were reluctant to offer HIVST to their partners if they had not disclosed their HIV-positive status to them; (3) there was limited use of strategies to support the disclosure of HIV status.

**Conclusion:** It is essential to strengthen strategies to support the disclosure of HIV+ status. It is necessary to develop a specific approach for the provision of HIV self-tests for the partners of PLHIV by rethinking the involvement of stakeholders. This approach should provide them with training tailored to the issues related to the (non)disclosure of HIV status and gender inequalities, and improving counseling for PLHIV.

## Introduction

For people living with HIV (PLHIV), HIV testing is the entry point for receiving life-saving treatment and care. HIV testing remains a pillar of HIV responses, as it also enables those testing negative to link to appropriate HIV prevention services. In 2019, 81% of PLHIV worldwide knew their HIV status; this proportion was estimated to be only 64% in West Africa (1). Such regional differences reflect difficulties in access to testing, which is related to stigma and discrimination against PLHIV (2). This fear of stigmatization causing difficulties related to the sharing of serological status in general and within couples has been reported in this area. This encouraged the establishment of support programs for the disclosure of HIV infection. However, few studies have been done to assess the impact of these programs (3–5).

To reach populations considered most vulnerable to HIV and with limited access to or uptake of conventional HIV testing services (which may be due to structural barriers), the World Health Organization (WHO) has recommended HIV self-testing (HIVST) since 2016 as a complementary approach. HIVST is defined as the process by which a person takes his or her own sample (oral fluid or blood); performs a test; and then interprets the results, often in a private setting, alone or with a trusted person (6).

In Eastern and Southern Africa, the HIV Self-Testing Africa Initiative (STAR), which pioneered the distribution of self-tests in this region, has tested different community-based delivery channels (door-to-door, within couples, among key populations, etc.) (7–9). Studies in other regions of Africa have also supported the efficacy, ease of use, and acceptability of HIVST (10–16).

Despite the high level of acceptability of HIVST, there has been little interest in couple testing, particularly among men. Two studies in Malawi and South Africa showed that men usually fear being in a serodiscordant relationship or being judged on their faithfulness (17, 18). A study conducted in Uganda among pregnant women showed the feasibility and effectiveness of HIVST secondary distribution to reach their male partners (i.e., giving self-tests to a pregnant woman to distribute to her partner). The study also emphasized the importance of support to minimize the risk of adverse effects such as violence or relationship breakdown (19).

Following STAR, the *AutoTest-VIH, Libre d'accéder à la connaissance de son Statut* (ATLAS) program was launched in West Africa with the objective of distributing nearly half a million HIV self-tests from 2019–2021 in Côte d'Ivoire, Mali, and Senegal. This program was initiated by a consortium composed of the non-governmental organization (NGO) *Solthis* and the *Institut de recherche pour le développement* (IRD). The ATLAS program introduced HIVST as an additional strategy in West Africa and was charged with organizing distribution, integration and scaling-up into national systems. The delivery of HIV self-tests was implemented through eight delivery channels and priority populations. Implementation plans were developed with country stakeholders (national AIDS programs/councils; international institutions, including the WHO; international and national NGO involved in local HIV programs; civil society; and community representatives). The priority groups include members of key populations (sex workers, men who have sex with men, and drug users), patients with sexually transmitted infections (STIs), and partners of PLHIV.

The ATLAS program integrates several research activities already described in detail elsewhere (20). This research component aims to generate and disseminate knowledge for the three countries and the West African region more broadly. The ATLAS program includes two qualitative studies conducted in Mali and Côte d'Ivoire to improve our understanding of the practices, limitations and issues related to the distribution of HIV self-tests to PLHIV for their partners. In these studies, “partner” is defined in a broad sense, i.e., regular or occasional, recent or former, formal or informal, and cohabitating or non-cohabitating. This article focuses only on the data from the first study conducted in Mali in an HIV care clinic in Bamako. Findings of the whole study, examined from an anthropological lens, will be published afterward.

The overarching aim of this study was to improve our understanding of the practices, limitations and challenges related to the distribution of HIV self-tests to PLHIV for partner testing in Mali. The estimated national HIV prevalence was 1.2% in 2019, and only 43% of PLHIV knew their HIV status (1). The rate of partner notification and disclosure of serological status is low in Mali, with an estimated 42% of PLHIV not having shared their HIV status with their partners in 2019 (21). In the next sections, we present the results from a qualitative study conducted in Bamako, the economic and political capital city of Mali.

## Materials and Methods

### Study Framework

The study was conducted in Bamako in a community HIV clinic with an active caseload of several thousand HIV patients (adults and children), more than two-thirds of whom were women. This clinic has good experience in community support. Since 2010, this clinic has been hosting a community empowerment program (Gundo_So: “Room of secrets” in Bambara) for women living with HIV to help them make informed choices about the disclosure of their HIV status to reduce the burden of HIV secrecy (22–24).

The clinic receives ~100 patients a day who pass through the reception service, which is then responsible for sorting and orienting patients according to the purpose of their visits.

#### Medical Consultations

Medical consultations are provided by two and four health professionals according to their availability. Consultations are not loyalty-based; i.e., a patient can be taken care of by any health professionals according to their availability. The reasons for consultation are diverse and include prescription renewal, follow-up check-ups and, rarely, consultation for not HIV related. Most medical consultations take place between 8:00 a.m. and 2:00 p.m. and are generally very short, lasting an average of 5 min, with most patients coming to renew their prescriptions. In some cases, the patient delegates a close person to pick up the medication for him or her.

#### Social Service Activities

Social service activities revolve around the psychosocial follow-up of patients and the general screening of people who have been referred by another health facility or who have presented for voluntary testing (pre- and post-test counseling, disclosure of results, etc.). The social worker is assisted by a peer educator for HIV testing and psychosocial follow-up activities. The social workers receive an average of 10 to 12 patients per day. Interviews can last between 10 and 30 min depending on the reason for the visit (HIV testing, psychosocial follow-up, etc.).

#### Group Discussions

Every Friday, cooking events are organized, which include group discussions (talks) facilitated by peer educators. They are attended by ~40 participants, two-thirds of whom are women. Talks take place before meals and last an average of ~30 min.

#### Introduction of HIVST

Following the example of Côte d'Ivoire and Senegal, in July 2019, the Solthis implementation team in Mali organized training sessions that aimed at imparting knowledge on strategies and methods of HIVST distribution before the introduction of HIVST. The training focused on the role of health professionals and the practical aspects of HIVST distribution in the ATLAS project. All the health professionals who were trained received the necessary materials for HIV self-test delivery. These materials included descriptive brochures for the demonstration/use of the self-test kit and the promotion of confirmatory testing and shareable video support (Youtube/WhatsApp) in French and translated into the main local languages (Bambara and Peul/Fula).

### Data Collection

We adopted a qualitative method combining observations and interviews (25–27). Data collection was carried out between September and November 2019 at the very beginning of the HIVST distribution in the facility, which started in August 2019.

The data were collected through (i) semi-structured individual interviews with health professionals who were directly or indirectly involved in HIV self-test distribution; (ii) observations of the clinical consultations of PLHIV; and (iii) observation of peer educator (psychosocial counselor)-led group talks attended by PLHIV. The data collection was carried out by the first author, who is an anthropologist, and the second author, who acted as the research assistant and interpreter (French-Bambara-French).

#### Semi-structured Interviews With Health Professionals

Individual interviews with health professionals were conducted in French using semi-structured interview guides (see Supplementary Table 1). Open questions were asked on these topics: introduction to HIVST, organization of the distribution of HIV self-tests, and practices and perceptions related to HIVST.

#### Observations of the Consultations

Consultations' observations included the consultations of PLHIV with two physicians, the consultations with a nurse prescribing antiretroviral drugs and the consultations with the social service office. These were routine consultations. The anthropologist and the research assistant attended consultations with various health professionals. Using an observation grid (see Supplementary Table 3), they observed exchanges between health care professionals and patients, noting their attitudes and the content of exchanges and specifically targeting attitudes and content related to HIVST. For all patients, they collected only age range and gender. For 51 patients whom HIVST was offered or discussed, they also collected marital status in addition to age range and gender. For the analysis, we only used observation reports and data from the 51 patients.

#### Observations of Group Discussions

The group discussions facilitated by peer educators involved bringing together the PLHIV who attended the clinic for a meal to discuss the benefits of local food. On this occasion, participants discussed various topics related to HIV. The discussions were conducted in Bambara (the most widely spoken local language in Bamako). We observed how the issues of HIV, AIDS, and HIVST were addressed by the facilitators and the reactions of the participants using an observation grid (see Supplementary Table 2).

We positioned ourselves as observers during steps (ii) and (iii) and avoided any intervention. The aim was to see how HIVST was approached by the facilitators and the participants.

### Collected Data

We conducted 8 individual interviews with health professionals whose characteristics are summarized in Table 1. Six of the eight health professionals interviewed benefited from ATLAS' training sessions. The other two health professionals (both peer educators) did not benefit from specific training on HIVST but had training on HIV testing. HIV self-tests were provided by only two physicians, the nurse and the social worker. The third physician, who was the clinic coordinator and was responsible for supervising HIVST activities, was not involved in the distribution of HIV self-tests “due to a lack of time,” according to his terms. The pharmacist oversaw the stock of HIV self-tests. The two peer educators addressed the issue of HIVST during the talks and group discussions that they delivered during cooking activities called “community meals.”

**Table 1:** Profile of the health professionals, including peer educators, who participated in the survey.

**Code**	**Category**	**Education level**	**Distributed HIV self-tests**	**Sex**
Health professional 1	Social worker	High	Yes	Female
Health professional 2	Physician	High	Yes	Male
Health professional 3	Physician	High	Yes	Female
Health professional 4	Nurse	High	Yes	Male
Health professional 5	Psychosocial adviser (peer educator)	Secondary	No	Male
Health professional 6	Psychosocial adviser (peer educator)	Secondary	No	Male
Health professional 7	Physician	High	No	Male
Health professional 8	Pharmacist	High	No	Male

We observed 556 medical consultations with the two physicians and the nurse, 35 consultations at the social service office, and seven group discussions. We toured the offices of the health personnel in charge of the dispensing of HIV self-tests for 1–5 days per office or according to the professionals' availability.

Profiles of the participants with who HIVST was discussed and/or proposed during the consultations are presented in Table 2. Among the 51 patients, age between 21 and 55, with whom HIVST was discussed and/or proposed during the consultations, the majority were women (42 women/nine men); 36 were married (31 women/5 men); six were in a relationship (three women/three men); three were single (two woman/one man); and five were widows (all women), only one declared have a partner.

**Table 2:** Profile of the participants with who HIVST was discussed and/or proposed during the consultations.

**N^**°**^**	**Sex**	**Marital status**	**Age range**
1	Female	Married	31–35
2	Female	Married	31–35
3	Female	Married	36–40
4	Female	Married	31–35
5	Female	Married	31–35
6	Female	Relationship	21–25
7	Female	Widow	31–35
8	Female	Married	31–35
9	Female	Married	31–35
10	Female	Married	41–45
11	Female	Relationship	21–25
12	Female	Widow, single	51–55
13	Female	Relationship	36–40
14	Female	Married	31–35
15	Female	Married	41–45
16	Female	Married	41–45
17	Female	Married	31–35
18	Male	Married	51–55
19	Female	Single	51–55
20	Female	Married	41–45
21	Female	Married	51–55
22	Male	Married	41–45
23	Male	Single	16–20
24	Female	Married	36–40
25	Female	Married	31–35
26	Female	Married	31–35
27	Male	Relationship	31–35
28	Female	Married	41–45
29	Female	Married	36–40
30	Female	Married	41–45
31	Female	Married	51–55
32	Female	Married	51–55
33	Female	Married	41–45
34	Female	Married	31–35
35	Female	Married	26–30
36	Female	Married	31–35
37	Female	Widow, single	41–45
38	Female	Widow, single	41–45
39	Female	Widow, single	51–55
40	Female	Married	31–35
41	Male	Married	41–45
42	Female	Single	21–25
43	Female	Married	41–45
44	Female	Married	36–40
45	Female	Married	51–55
46	Male	Married	51–55
47	Female	Married	21–25
48	Male	Married	31–35
49	Male	Relationship	41–45
50	Male	Relationship	31–35
51	Female	Widow, in a relationship	36–40

### Data Analysis

#### Interview Analysis

All interviews with health professionals were recorded with their consent. Then all recorded interviews were transcribed before being coded and analyzed using Dedoose qualitative data analysis software (https://www.dedoose.com/). The codes and subcodes were defined based on the themes developed in the interview guides and then refined based on the content analysis of the data (28, 29).

#### Observation Analysis

Observation notes taken during group discussions were subjected to content analysis. Regarding the observation notes taken during consultations with health professionals, only those where HIVST was discussed and/or proposed were considered in the content analysis (*n* = 51).

Data analysis was based on a Grounded Theory approach i.e., a theory developed by induction from a corpus of data (30). A gendered approach was used to account for the effects of gender on HIVST in the data analysis (31).

### Ethical Approvals

The study protocol, including consent sheets and procedures, was approved by the WHO Ethical Research Committee (07 August 2019, reference: ERC 0003181), the National Ethics Committee of Life Sciences and Health of Côte d' Ivoire (28 May 2019, reference: ERC 0003181): 049-19/MSHP/CNESVS-kp), the Ethics Committee of the Faculty of Medicine and Pharmacy of the University of Bamako, Mali (August 14, 2019, reference: 2019/88/CE/FMPOS), and the National Ethics Committee for Health Research of Senegal (July 26, 2019, protocol SEN19/32).

## Results

In this section, we first describe how the issue of HIVST is addressed in medical consultations, social service consultations, and group discussions. Next, we present the reasons why it is difficult for health professionals to discuss or propose HIVST during consultations. Finally, we present three barriers to the proposal of HIVST when disclosure of HIV status is not done within the couple.

### Approach to HIVST

The approach to or presentation of HIVST differed from one health professional to another, even if all health professionals used the same tools (HIVST kits with instructions for use and videos describing how to use HIVST). Two of the health professionals (one physician and one nurse) used both the video in Bambara and the instructions to present HIVST to PLHIV, while the other two (one social worker and one physician) relied only on the instructions.

The two peer educators, on the other hand, only showed the HIVST kit without going into much detail about its use.

#### HIVST Was Discussed in Almost All Talks (and Group Discussions)

The issue of HIVST was raised in almost all the talk sessions (6/7) that we attended, either to provide information, to remind the participants about the existence of HIVST or to invite the participants to talk about HIV self-tests or to propose testing to their partners.

“*I remind you that we discussed the existence of a new screening technique here. We use it in the mouth”* (from a peer educator).

Mentions of HIVST during the discussions were always followed by exchanges with and questions from the participants about how the tests are used, especially how they could be offered to partners when one's HIV status had yet to be disclosed with one's partner.

#### Discussion About HIVST Increased With the Length of Medical Consultations

Consultations during which HIV self-tests were provided lasted between 10 and 30 min depending on the health professionals and how HIV self-tests were offered to patients for their partners' use. The two health professionals (one physician and one nurse) who used both video and the paper instructions to explain how to use HIVST spent an average of 20–30 min per person, while the other two (social worker and 1 physician) who used only the instructions spent less time (10–15 min). Medical consultations lasted an average of 5 min, while social service consultations lasted from 10 to 30 min.

We did not notice any difference in the way HIV self-tests were delivered according to the gender of the health professional. Exchanges between health professionals and patients were conducted mostly in Bambara and rarely in French. During the 591 observed consultations, of which ~35 were at the social service office, most PLHIV who presented were women (*n* = 450/591, 76%).

Proposing HIVST could affect health professionals' workload, especially during medical consultations. In some cases, the proposal of HIVST considerably lengthened waiting times, and patients complained about the delays. Although such delays were not explicitly mentioned by the health professionals responsible for the delivery of HIVST as one of the reasons for the low rate of HIVST proposal, they nonetheless represented a non-negligible factor.

*Interviewer: How long do you do [a consultation]?*

*Health professional: It is more than 30 min anyway*.

*Interviewer: ounhoun (ok). When you have to show the video, ehh*.

*Health professional: You have to explain. You have to show the video; you have to explain. It takes at least 30 min*.

*Interviewer: But does it change anything in your work? Is it additional work?*

*Health professional: Yes, yes. Because at the very beginning, it can often lead to a slowdown in your work. (.) often the sick will yell out there*.

*(Interview with a health professional)*.

#### Difficulties Discussing HIVST During the Observed Consultations

The results showed that there were difficulties for health professionals in discussing or proposing HIVST during consultations because, prior to the introduction of HIVST, the discussion of HIVST was deemed inappropriate for most consultations and some PLHIV had not disclosed their HIV status to their partners.

Indeed, according to our observations and the explanations of the health professionals in charge of providing HIVST, the following two situations could lead health professionals to avoid discussing the issue of HIVST or proposing that PLHIV give the HIVST kit to their partners:

1 It was not appropriate to dispense an HIV self-test at the consultation: e.g., if a patient with HIV was represented by a third party to renew his or her prescriptions or if the health professional knew in advance or after questioning the patient that he/she was widowed or single (without a partner) or that his or her partner was already receiving care, the issue of HIVST was not generally addressed or was just briefly mentioned.

*Excerpts from exchanges between patients and health professionals during consultations where HIV self-tests were not provided:*

*Extract n*°*1:*

*Health professional: Do you have a partner(s)?*

*Patient: No*.

*Health professional: The reason I asked you this is because there is a new at-home test. It is in the experimental phase*.

*Patient: Ok, it is (.)*

*(Extract from an exchange between a health professional and patient during a consultation)*

*Extract n*°*2:*

*Health professional: Has your husband been tested?*

*Patient: Yes. He is even followed here at the XXX (clinic)*.

*Health professional: The reason I am asking you this is because we now have a way for people to test themselves for HIV/AIDS*.

*Patient: Ok, I heard*.

*(Extract from an exchange between a health professional and patient during a consultation)*

2 When the health care professional knew that the patient with HIV had not disclosed his or her status and did not wish to do so, the health care professional generally avoided offering the patient an HIVST kit for his or her partner, considering, e.g., that it might “be complicated.”

*Health professional: Are you married?*

*Young man: No*.

*Health professional: Do you have a sexual partner(s)?*

*Young man: Yes, I do*.

*Health professional: Has she been screened?*

*Young man: Not yet. She will do it when we are engaged*.

*Health professional: Have you shared your status with her?*

*Young man: Not yet*.

*Health professional: Ok. We have a test for that, which is done in the mouth. But since you haven't shared your status yet, it's going to be complicated*.

*(Excerpt from an exchange between a patient and a health professional during a consultation where HIVST was not dispensed)*

Overall, HIVST was discussed during only 51 [42 women (W) and nine men (M)] of the 591 observed consultation (9%); in 49 of the consultation, the health professional initiated the discussion on HIVST, and PLHIV initiated the discussion two times. In the 49 consultations (40 W and nine M) where the discussion was initiated by the health professional, six PLHIV (5 W and 1 M) were found not to have a partner after the discussion, five (4 W and 1 M) had partners who had already been tested or followed up for HIV, 27 (22 W and 5 M) had disclosed their HIV status to their partners and 11 (9 W and 2 M) had not disclosed their HIV status to their partners. A total of 37 proposals for HIVST were made to PLHIV, of which 28 proposals (23 W and 5 M) were accepted and 9 were refused (8 W and 1 M).

### The Three Main Barriers to the Distribution of HIV Self-Tests

The observations of the consultations and focus group discussions with peer educators and interviews with health professionals revealed three main barriers to the distribution of HIV self-tests in the context of low HIV status disclosure.

#### Health Professionals Avoided Offering HIVST to PLHIV Who Did Not Have Partners or Did Not Want to Disclose Their HIV+ Status to Their Partners

During the interviews, the four health professionals in charge of providing HIV self-tests considered the disclosure of one's HIV status to be a prerequisite for offering testing (and thus for providing HIV self-tests) of partners of PLHIV, as illustrated in this excerpt from an interview with a health professional.

*Health professional: (…) it is people who are monitored at the clinic level and who wish to screen their partners. Now, it would be necessary that, first of all, the person shares his status*.

*Interviewer: Ounhoun (ok)*.

*Health professional: If not, the person is offered to share [his or her HIV status with his or her partner]. Because you can't just give the test to someone who may not have shared their status*.

*Interviewer: Ok. So that's been said since the training*.

*Health professional: No, no, no. In practical terms*.

*Interviewer: But in relation to training, they didn't exclude this case for example?*

*Health professional: No, no, no*.

*Interviewer: A person who hasn't shared [his or her status], we can't offer the test? (…)*

*(Interview with health professional)*

In our observations, the four health professionals who distributed tests avoided offering HIV self-tests for index testing when they knew that the individual's HIV status had not been disclosed to his or her partner. In two instances, the proposal of HIVST was withdrawn when the health professional realized that the patient had not disclosed his or her status—as described in the excerpt below from an observation note from a medical consultation.

*Health professional: Has your husband been tested?*

*Woman: He is not infected*.

*Health professional: Has he been tested or not?*

*Woman: Yes, he did, and he renews it every 3 months*.

*Health professional: Okay. The reason I am asking is that we now have a “test” (referring to the self-test kit). It's just done with saliva, and the result can be read after 20 minutes*.

*Woman: Ok*.

*Health professional: You could bring it to him for home testing. I will show you a video explaining how to use it in Bambara*.

*Woman: Ok*.

*Health professional (after viewing): Did you understand?*

*Woman: Yes. Could I do it too?*

*Health professional: No. Those who are already HIV-positive are not allowed to do it. Only your husband could do it*.

*Woman: Okay. But I haven't shared my status with my husband yet*.

*Health professional: Then, it's going to be complicated because he might ask you questions about where the kit comes from. What could you say in that case?*

*Woman: Oh, that's right. I hadn't thought of that*.

*Health professional: Even if you told him it came from a hospital, he might ask you to specify which one?*

*Woman: Yes, that's right*.

*Health professional: Or are you going to share your status with him now?*

*Woman: Not at all. [He is] someone who already wants to divorce me; I'm not going to add more*.

*Health professional: Ok, I understand. Then I won't be able to give you a self-test kit anymore*.

*Woman: Ok*.

*(Extract from an exchange between a health professional and patient during a consultation)*.

#### PLHIV Were Reluctant to Offer HIV Self-Tests to Their Partners If They Had Not Shared Their HIV Status

Despite the position of health professionals that disclosing one's HIV status to a partner was a prerequisite for offering an HIV self-test for the partner, offers for HIV self-tests were indeed made to people who had not shared their HIV status and did not wish to do so. Of the 27 people who had already shared their status and were offered HIVST, 26 (21 W and 5 M) agreed to give HIV self-tests to their partners, while of the nine who had not shared their status, seven (6 W and 1 M) refused to so, with six (5 W and 1 M) explicitly mentioning or implicitly implying non-disclosure as the main reason for refusal. This finding shows that disclosure of one's HIV status to one's partner is a determining factor in the acceptance of the proposal of partner HIVST by PLHIV.

*Example 1*

*Health professional: Is your husband here?*

*Woman: Yes, he is at home*.

*Health professional: Is he under treatment?*

*Woman: No*.

*Health professional: Did you share your status with him?*

*Woman: No. I am very afraid*

*Health professional: Okay. But are you going to tell him one day?*

*Woman: No (while lowering her head)*.

*Health professional: Why? And yet you've been followed here for 14 years*.

*Woman: I'm very scared. I would like him to find out from me one day, but I am very afraid*.

*Health professional: Okay. But it can't go on like this. You can't keep it from him forever*.

*Woman: Yes, I know that*.

*Health professional: If you were given something, could you send it to him for testing?*

*Woman: No, I can't (with her head down)*.

*Health professional: So he's going to ask you if you did it too?*

*Woman: Yes*.

*(Extract of health professional/patient exchange during a consultation)*

*Example 2*

*Health professional: Have you shared your status with your husband?*

*Woman: No*.

*Health professional: Why?*

*Woman: Because he's going to tell everyone (.)*

*Health professional: I still advise you to think about it since it would be better if he were to be screened and even followed up if necessary*.

*Woman: Yes, that's right*.

*Health professional: Otherwise, we have a way for him to do it [the test] at home*.

*Woman: No, it's ok*.

*(Extract of health professional/patient exchange during a consultation)*.

#### Strategies to Support the Disclosure of HIV Status Had Limitations

The third barrier was related to the limitations of existing support strategies for disclosing HIV-positive status when proposing HIVST to PLHIV who had yet to disclose their status to their partners. Despite the presence of a support program for disclosure called *Gundo-So* in the facility, health professionals and patients often felt powerless to overcome barriers to disclosure.

In the interviews, the health professionals acknowledged that the *Gundo-So* program is useful but has limitations regarding inclusion criteria, particularly in terms of timing (only women who have discovered their HIV status within the last 6 months to 5 years can participate). Moreover, this program, based on values of autonomy and empowerment, is not intended to force women to disclose their HIV status, which could in some cases put them at risk, but rather to accompany them in their choice of whether to share their status.

“*Gundo-so” does exist. But the “Gundosso” doesn't intervene directly to tell someone to share “(.) They didn't want to take everyone. They wanted to take people who have disclosed recently (6 months to 5 years)*.

*(Interview with one health professional)*

In the peer educator-led group discussions for PLHIV, one of the first requests from patients after the mention of HIVST was tips on how to offer HIV self-tests for index testing without having to disclose their HIV status.

In the two consultations in which HIV self-tests were accepted by patients who had not yet disclosed their status to their partners, the health professional left it up to the patients to manage the disclosure themselves and did not offer them any specific support. Consequently, one patient proposed a strategy by requesting two test kits so that she and her partner could test simultaneously (i.e., without having to disclose her serological status prior to the test). The health professional accepted the request, telling the patient that the HIV self-test would probably be “indeterminate” [viral load undetectable with antiretroviral (ARV) treatment]. During the ensuing discussion, the young patient seemed very hesitant and anxious about offering her partner the HIV self-test. According to her, her partner was very smart and would certainly ask where she had received the test and why she did not test herself at the same time as him. The health professional explained to us that the patient's case was somewhat unique since she could not use the HIVST kit since she was already HIV positive and on ARV treatment. On the other hand, the health professional said he was obliged to give her the two HIVST kits since the patient considered this to be the only way for her partner to agree to be tested. After showing the video, the physician continued as follows:

*Health professional: Did you get the message?*

*Woman: Yes*.

*Health professional: So he should be convinced to do it*.

*Woman: It's not going to be easy. He will ask me to use it first. So I would need two kits for that*.

*Health professional: Ok. I will give you two kits, but you should know that your result will be insignificant for us*.

*Woman: Ok*.

*Health professional: You'll know how to do it, right?*

*Woman: Yes (smiling)*.

*(Excerpt from exchanges between physician and patient)*

## Discussion

Using qualitative survey methods, we found several difficulties for health professionals to propose HIVST to their patients and for PLHIV to accept the proposal for index testing in the context of the high rate of HIV non-disclosure within couples. Specifically, we identified three main barriers to the provision of HIVST for index testing. First, almost all health professionals avoided offering HIV self-tests to PLHIV when they thought or knew that PLHIV had not shared their HIV+ status with their partners or did not wish to do so. Second, PLHIV were reluctant to offer HIV self-tests to their partners if they had not disclosed their own HIV+ status. Third, it was difficult for health professionals and PLHIV to manage the offer of HIVST and the disclosure of HIV+ status with the partner.

### Difficulties in Proposing HIVST for the Partners of PLHIV Were Exacerbated by the Non-disclosure of HIV Status

The difficulties of discussing or proposing HIVST to PLHIV for health care staff notably resulted from the fact that most consultations were not appropriate for HIVST proposal to partner (e.g., when PLHIV were widowed, did not have a partner, or had delegated someone to renew their prescriptions). In addition, health care professionals were reluctant to discuss HIVST with their patients when they knew that their patients had not disclosed their HIV+ status with their partners. Other factors, such as the time-consuming nature of dispensing HIV self-tests, should not be overlooked among the underlying reasons for the low proportion of HIV self-tests dispensed in consultations.

The fears of PLHIV regarding the possible adverse consequences following the disclosure of HIV-positive status and the difficulties of health professionals in supporting PLHIV in this process were identified in this study as important barriers to the secondary distribution of HIV self-tests for index testing. In West Africa, the difficulties of disclosing HIV+ status to a partner results from a structural problem related to low self-esteem and fear of stigmatization or rejection by the partner, especially among women (32–35). Studies conducted in Malawi and Uganda on testing within couples at home attributed the low use of HIVST, especially among men, to a fear of having one's infidelity revealed, absence from home due to their professional activities, and fear of marital breakdown (18, 36). In Burkina Faso, an analysis of the effects of gender on testing showed that while fear of rejection by partners, friends or family members was cited as a reason for not using testing in general, women also cited a fear of losing their livelihoods (37). A woman's precariousness and/or financial dependence is a factor that reinforces her vulnerability to the undesirable effects of sharing HIV status within the couple (38). For this reason, a study conducted in Mali as part of the *Gundo-So* program emphasized the need to strengthen programs supporting PLHIV and empower PLHIV so that they can make free and informed decisions regarding the disclosure of their HIV status (22).

### HIVST: A Limited Opportunity for Status Sharing and Partner Testing

HIVST could be seen as an opportunity for PLHIV to disclose their status to their partners. Surveys of same-sex couples in China and South Africa found an increase in the disclosure of HIV status with the partner before having sex with each other as a result of access to HIV testing (17, 39). However, this finding may be specific to the marital context and the nature and duration of those relationships. We did not find any specific study that documented the link between access to HIVST and disclosure of HIV status among PLHIV.

The ATLAS project promotes HIVST for the partners of PLHIV regardless of disclosure status, considering that HIVST could represent an opportunity to facilitate the disclosure process (and thus reduce barriers to access to testing, such as coming to the health center). However, the project recognizes the importance of assisted notification to promote partner testing and has therefore integrated these elements into the definition of dispensing strategies, training programs and tools available to dispensing agents. e.g., one of the key message of the training course was: “Assisted partner notification improves uptake of testing and is a simple and effective way to reach partner of PLHIV.” (https://atlas.solthis.org/wp-content/uploads/2019/11/03_Manuel_Formateur_ProSante_M3_ML.pdf) (see Supplementary Table 5).

However, in this study, while the ATLAS project did not define the notification of one's own HIV status to one's partner as a condition of the proposal of partner HIVST to PLHIV, disclosure was often considered a prerequisite by the health professionals and by some PLHIV. The hesitance of health care professionals and patients regarding the proposal of HIVST could be interpreted as a desire to anticipate possible adverse effects in couples that are not always justified (40).

Furthermore, the attitude of the patient who requested two HIVST kits to be able to carry out couple testing without having to disclose her HIV+ status and the acceptance of the request by the physician who informed her the result would be “insignificant” (a false negative) raise an ethical issue which could be analyze.

It is essential to strengthen strategies to support HIV+ status disclosure, the HIV testing of PLHIV partners, and the development of anti-stigma programs to improve HIV+ status disclosure and the uptake of HIV testing in general. Experiences with couple testing strategies, especially in the context of the prevention of mother-to-child transmission, could be mobilized to reach more untested partners. Indeed, the effectiveness of couple-based testing approaches and support for women has been demonstrated in numerous studies (41–43).

In Mali, comprehensive testing of HIV-positive partners cannot be effective without improving support for the disclosure of HIV+ status by strengthening “couples” counseling that takes into account the gendered dimensions of disclosure. However, despite the existence of a program to support the disclosure of HIV status like *Gundoso*, the impact of this type of intervention on the sharing of status within the couple has hardly been documented, as in other sub-Saharan countries as noted by systematic reviews (3–5). Also, improving support would involve the consideration of programs to support women's empowerment (22, 44).

### Delegation of Tasks: An Opportunity to Improve the Distribution of HIVST

In addition to the non-disclosure of serological status, there were difficulties with HIVST distribution since most of the information and distribution of HIV self-tests to PLHIV for index testing was carried out by medical staff who were already overwhelmed by “normal consultations,” which could hinder the distribution of HIV self-tests (45). Increasingly, however, task shifting seems to be a preferred option in the monitoring and support of PLHIV because it has proven its worth in the response to HIV (23). In the context of the introduction of HIVST, particularly through index testing, the involvement of non-medical staff such as social workers, peer educators or other community actors could promote better distribution because these non-medical staff have much more time for exchange with patients and/or proximity with patients, which would reduce the cost of dispensing HIVST (45).

### Limitations of the Study

The study was conducted on one site. The results of this study rely on data collected only 3 months after the start of HIVST dispensing activities in Mali. Additional interviews and observations in the same facility are planned before the end of the project in 2021 to document any changes related to the provision of HIV self-tests.

## Conclusion

The difficulties of offering HIVST to partners of PLHIV raise fundamental questions related to HIV disclosure to sexual partners and the associated stigmatization. Our results highlight the potential role of interventions to support HIVST for index testing that does not rely on disclosure and that is adapted to local contexts to increase diagnostic coverage of partners of PLHIV who are not reached by traditional testing strategies.

It is necessary to develop a specific approach for the provision of HIV self-tests for the partners of PLHIV by rethinking the involvement of stakeholders (caregivers, social workers, peer educators, etc.). This approach would involve reviewing the roles assigned to these stakeholders, providing them with training tailored to the issues related to the disclosure or non-disclosure of HIV status and gender inequalities, and improving counseling for PLHIV regardless of their situations.

## Data Availability Statement

The original contributions presented in the study are included in the article/Supplementary Material, further inquiries can be directed to the corresponding author/s.

## Author Contributions

JL, AD, DP, and NR designed and implemented the ATLAS STUDY. SBoy, DP, and JL conceived and designed the analysis. SBou and SBoy collected the data. SBoy wrote the first draft of the manuscript. All authors contributed to the interpretation and presentation of the findings and approved the final version of the manuscript for submission.

## Conflict of Interest

The authors declare that the research was conducted in the absence of any commercial or financial relationships that could be construed as a potential conflict of interest.
