# Supplementary material for: Challenges of HIV Self-Test Distribution for Index Testing When HIV Status Disclosure Is Low: Preliminary Results of a Qualitative Study in Bamako (Mali) as Part of the ATLAS Project
Source: Front Public Health. 2021 May 19;9:653543. doi: 10.3389/fpubh.2021.653543 (PMC8170018; doi:10.3389/fpubh.2021.653543)
Supplement: Supplementary file 1 [file Table_1.pdf]

Thematic interview guide - Healthcare workers

General Information

Age

Vocational training

Workplace

Professional activity

Involvement in the care of people living with HIV/AIDS

Description of the service's activities

HIVST and screening of partners of PLHIV

Before the introduction of HIVST: position/activities of the service in relation to the screening of PLHIV partners, support for PLHIV in sharing their status with the partner.

Date and progress of the implementation of HIVST for PLHIV partners: inconveniences, obstacles, tools mobilised to implement HIVST for PLHIV partners, adaptation of consultations, information mobilised, choice of PLHIV to whom HIVST is proposed for their partner...

Confidentiality management

Attitudes of PLHIV towards HIVST's proposal for their partners

Content of the information presented to them (describe the terms used to present HIVST to them)

Refusal / acceptance / doubt / questions

Attitudes of PLHIV and/or partners after use of HIVST

Responses of PLHIV and partners after testing based on the results (do they inform about the results? How? (By telephone; face-to-face; accompanied; indirect information via partner) time frame)

Support for partners with a positive HIVST result

Confirmation of status after TA screening

Role of the PLHIV in the support of its partner

Care-giver-care relationship when screening was done by HIVST
