# Supplementary material for: Challenges of HIV Self-Test Distribution for Index Testing When HIV Status Disclosure Is Low: Preliminary Results of a Qualitative Study in Bamako (Mali) as Part of the ATLAS Project
Source: Front Public Health. 2021 May 19;9:653543. doi: 10.3389/fpubh.2021.653543 (PMC8170018; doi:10.3389/fpubh.2021.653543)
Supplement: Supplementary file 2 [file Table_2.pdf]

Observation Guide - Meetings Sites

General information

Location

Type of activity

Moment

Duration

Persons present

Specific information about the activity

Objective of the activity/order of the day

Profile of the participants

Questions about HIV and AIDS

Are issues around HIV and AIDS addressed during staff meetings?

By whom?

How are they approached?

Participants' reactions

Positive/negative points discussed

Difficulties around the dispensary?

Negotiations on the terms and conditions of dispensation, the people to whom the self-test should be offered, the information to be provided, etc.

Discussions on HIV partner testing and how to provide self-tests to partners.

Discussions on confidentiality issues
