# Supplementary material for: Challenges of HIV Self-Test Distribution for Index Testing When HIV Status Disclosure Is Low: Preliminary Results of a Qualitative Study in Bamako (Mali) as Part of the ATLAS Project
Source: Front Public Health. 2021 May 19;9:653543. doi: 10.3389/fpubh.2021.653543 (PMC8170018; doi:10.3389/fpubh.2021.653543)
Supplement: Supplementary file 3 [file Table_3.pdf]

Observation Guide - HIV Consultation

General information

Location

Moment

Duration

Persons present

HIVST's proposal for the partner

Circumstances: "routine" or specific consultation

Information provided, terms used

Responses of PLHIV

Questions asked

Acceptance, refusal, request for time to think, more information?

Reasons expressed

When the HIVST kit is delivered

Description of the kit

Description of the information provided

Description of the questions asked / reactions of the patient

Social report

Terms and conditions of exchanges

Attitudes and gestures
